# Generating *Vegfr3* reporter transgenic mouse expressing membrane-tagged Venus for visualization of VEGFR3 expression in vascular and lymphatic endothelial cells

**DOI:** 10.1371/journal.pone.0210060

**Published:** 2019-01-02

**Authors:** Chisato Watanabe, Jun Matsushita, Takuya Azami, Setsuko Tsukiyama-Fujii, Tomoyuki Tsukiyama, Seiya Mizuno, Satoru Takahashi, Masatsugu Ema

**Affiliations:** 1 Department of Stem Cells and Human Disease Models, Research Center for Animal Life Science, Shiga University of Medical Science, Otsu, Shiga, Japan; 2 Animal Resource Center, University of Tsukuba, Tsukuba, Ibaraki, Japan; 3 Department of Anatomy and Embryology, Faculty of Medicine, University of Tsukuba, Tsukuba, Ibaraki, Japan; 4 International Institute for Integrative Sleep Medicine, Life Science Center, University of Tsukuba, Tsukuba, Ibaraki, Japan; Medical College of Georgia at Augusta University, UNITED STATES

## Abstract

*Vascular endothelial growth factor receptor 3* (*Vegfr3*) has been widely used as a marker for lymphatic and vascular endothelial cells during mouse embryonic development and in adult mouse, making it valuable for studying angiogenesis and lymphangiogenesis under normal and pathological conditions. Here, we report the generation of a novel transgenic (Tg) mouse that expresses a membrane-localized fluorescent reporter protein, *Gap43-Venus*, under the control of the *Vegfr3* regulatory sequence. *Vegfr3-Gap43-Venus* BAC Tg recapitulated endogenous *Vegfr3* expression in vascular and lymphatic endothelial cells during embryonic development and tumor development. Thus, this Tg mouse line contributes a valuable model to study angiogenesis and lymphangiogenesis in physiological and pathological contexts.

## Introduction

The vascular endothelial growth factors (VEGFs) play as critical regulators of vascular development among mammalian species [1]. The VEGF family consists of six secreted proteins (VEGF-A, B, C, D, E and placental growth factor), which have different binding affinities for three tyrosine kinase receptors; VEGFR1 (Fms-like tyrosine kinase 1; Flt1), VEGFR2 (Fetal liver kinase 1; Flk1), VEGFR3 (Fms-like tyrosine kinase 4; Flt4)[1,2]. VEGF family members also bind to non-tyrosine kinase receptors, Neuropilin1 and 2, which are considered to function as co-receptors for the VEGFRs. VEGF-A and VEGF-B and placental growth factor bind to VEGFR1, VEGF-A and VEGF-C to VEGFR2, and VEGF-C and VEGF-D to VEGFR3. VEGF-C binds to VEGFR2/VEGFR3 heterodimer and transduces signals through Akt, whereas VEGFR3/VEGFR3 homodimer transduces signals through ERK [3].

In adult tissues, VEGFR1 and VEGR2 are most strongly expressed and play a crucial role in vascular endothelial cells [4]. In contrast, VEGFR3 expression initially occurs in vascular endothelial cells, and then is maintained both in vascular and lymphatic endothelial cells, later becoming largely restricted to lymphatic endothelial cells [5]. However, VEGFR3 is induced again at a high level in vascular endothelial cells in adulthood during physiological and pathological angiogenesis [6], and blocking VEGFR3 with antibodies results in decreased angiogenesis during postnatal retinal development [7]. Other researchers have reported that Notch-dependent VEGFR3 up-regulation allows angiogenesis without VEGF/Flk1 signaling. VEGFR3 is also expressed in non-endothelial cells such as neural cells, macrophages and osteoblasts [8,9]. Thomas and coworkers showed that VEGFR3 is expressed in neural stem cells and its function is required for neurogenesis.

For better understanding the cellular and molecular mechanism of angiogenesis, we created animal models useful for visualization and live imaging of vascular endothelial cells expressing VEGFR1 or VEGFR2 [10,11]. For example, we generated *Vegfr2-GFP* BAC transgenic mice to monitor *Vegfr2* gene expression during development [10,12]. Although *Vegfr1* and *Vegfr2* are expressed in most endothelial cells during development, the expression levels are different in specific endothelial cells, such as tip and stalk cells in the developing retina, and their relative expression levels correlate with the position of the endothelial cells. In double transgenic mice expressing both *Vegfr2*-GFP and *Vegfr1*-tDsRed genes, significantly different expressions between *Vegfr2* and *Vegfr1* were observed in E9.5 embryos [11]. The *Vegfr2-GFP* :: *Vegfr1-tDsRed* double transgenic mice have been used by a number of laboratories [13].

Here, we report the generation of a novel fluorescent reporter mouse line expressing a membrane bound form of Venus, a bright fluorescent protein [14], under the control of the *Vegfr3* BAC (bacterial artificial chromosome) transgene to visualize VEGFR3 expression during embryonic development and in adulthood. Our results show the recapitulation of endogenous VEGFR3 expression governed by *Vegfr3-Gap43-Venus* BAC Tg in the vascular endothelial and lymphatic endothelial cells. Our result also shows that the Tg mouse is useful for illuminating the cell shape of endothelial cells owing to the advantage of membrane-tagged Venus. This will serve as a valuable tool for marking VEGFR3-expressing cells during development and in adulthood under normal and pathological conditions.

## Material and methods

### Constructing the *Vegfr3-Gap43-Venus* BAC Tg transgene

The BAC clone (RP23-210C22) encompassing the mouse *Vegfr3* gene was purchased from Invitrogen (Carlsbad, CA, USA). A DNA fragment encoding membrane localization signal of mouse *Gap43* (5’-ATGCTGTGCTGCATGCGAAGAACCAAACAGGTTGAAAAGAATGATGAGGACCAAAAGATC-3’) was ligated to a *Venus-bGH polyA* cassette and the resultant DNA was further ligated to an *FRT PGK-gb2 neo* expression cassette that comprises a prokaryotic promoter and neomycin-resistance gene flanked by FRT sequences (Gene Bridges, Heidelberg, Germany). Homology arms for the second exon of *Vegfr3* were amplified by two pairs of primers as follows: 5’arm-1: 5’-ATACTCGAGACGCGTGGCCTTGACCAGAGCAAGGAACAG-3’, 5’arm-2: 5’-ATAGAAGACCGGGGCCGGGGTTCTCCTC-3’, 3’arm-1: 5’-ATAGCGGCCGCGTATCCTGGCTCCTCCCCACCTG-3’, and 3’arm-2: 5’-ATAGGCGCGCCAGATCCACTCCCTGAGCCCTTGAC-3’. Afterwards, the arms were ligated to both ends of the reporter cassette. The resulting donor vector and RED/ET expression plasmid (Gene Bridges) were introduced into *Escherichia coli*. The recombinants were identified by PCR analysis. The neo cassette was excised by transient expression of FLPe (Gene Bridges).

### Generating Tg mice

Recombinant BAC DNAs for *Vegfr3* were purified with a NucleobondXtra BAC Kits (Macherey-Nagel, Düren, Germany) and then linearized by Pl-SceI digestion. Pronuclear injection was performed in fertilized eggs isolated from C57B6/J females (SLC Inc., Shizuoka, Japan). Tg mice were confirmed by PCR genotyping with the following primer sequences: GFP-S2, 5’-TTCAAGGACGACGGCAACTACAAGAC-3’ and GFP-AS2: 5’-GCTTCTCGTTGGGGTCTTTGCTCAG-3’. The Tg mice were maintained on ICR or C57B6/J genetic background. *Vegfr2-GFP* BAC Tg mice[10], *Vegfr1-tDsRed BAC Tg* mice[11], *Vegfr2+/GFP* knock-in mice[15] were described as previously.

The experimental procedures were approved by the ethics committee for Animal Experimentation of Shiga University of Medical Sciences (Approval number: 2016-2-8) (Committee member is Akira Andoh, Jun Udagawa, Masatsugu Ema, Kazumasa Ogasawara, Shinichiro Nakamura, Kazuhiko Nozaki, Seiji Hitoshi, Kihachiro Horiike, Yoshihito Muroji, Shigehiro Morikawa). Masatsugu Ema was not involved in the judgement. Implantation of LLC tumor cells was performed under isoflurane anesthesia.

### Immunohistochemical analysis

Embryos were then dissected, and fixed in 4% paraformaldehyde for 3 h at 4°C as described previously [16]. For whole mount immunohistochemistry, after washing twice in PBS, embryos were permeabilized in 0.5% TritonX-100 (Sigma-Aldrich, St. Louis, MO, USA) in PBS for 30 min at 4°C. Permeabilized embryos were then blocked in blocking solution containing 10% donkey serum (Immuno Bioscience, Mukilteo, WA, USA), 0.2% bovine serum albumin (Sigma-Aldrich) and 0.01% PBT (0.01% Tween20 in PBS, Nacalai Tesque, Inc., Kyoto, Japan) for 1 h at 4°C, followed by incubation overnight at 4°C. After three washes with 0.2% PBT, embryos were incubated with secondary antibodies, listed in S1 Table, for 3 h at 4°C. Embryos were then washed three times with 0.2% PBT and incubated with anti-GFP Alexa-Fluor488 conjugate (1:200; Cat #A21311, Life Technologies, Inc. Carlsbad, California) for 1 h at RT. Nuclei were stained with Hoechst33342 (10 μg/ml; Cat #H3570, Molecular Probes Inc.) for 20 min at 4°C. The first and second antibodies are listed in S1 Table.

For cryosections, embryos, adult back skin and tumors were mounted in OCT (Surgipath FSC 22 Blue Frozen section compound; Leica, Wetzlar, Germany) as described previously. Tissue blocks were sectioned using a cryostat (CM1860 UV; Leica). The sections were processed as described previously [16] and mounted by PermaFluor (Thermo Fisher Scientific, Waltham, Mass). For staining of cortex at E14.5 and E17.5, 100μm coronal sections were stained by the procedure for whole mount embryos.

### Confocal microscopy

Images of embryos were captured by Leica MZ FLIII with a GFP LP filter (Leica Microsystems, Wetzlar, Germany). Confocal images were acquired on a Leica TCS-SP8 (Leica). Embryos were mounted in PBS on glass-bottom dishes (IWAKI, Tokyo, Japan). Fluorescence was excited with a 405-nm UV laser for Hoechst 33342, a 638-nm laser for Cy5 or Alexa Fluor 633, a 552-nm laser for Cy3, and a 488-nm laser for Alexa Fluor 488. The hybrid detector HyD (Leica) was used for signal detection. 3D reconstruction was performed by 3D viewer software (Leica) from z-stack images every 5 μm.

### Quantitative colocalization analysis

Images acquired from confocal microscopic analysis were processed by NIH ImageJ FIJI version 2.0.0-rc-65/1.5/w software [17]. In brief, RGB color images were converted into grey scale images and Pearson’s *R*-values (above thresholds) were calculated and presented as a 2D intensity histogram.

### Western blotting

E9.5 embryos dissected from *Vegfr3-Gap43-Venus* BAC Tg mice were treated with 0.25% trypsin (Thermo Fisher Scientific) for 5 min at 37°C and Venus-positive cells were sorted by a FACSAria Fusion instrument (BD Biosciences, Franklin Lakes, NJ, USA). The sorted Venus-positive cells were suspended in RIPA buffer (50 mM Tris-HCl, 150 mM NaCl, 1 mM EDTA (pH 8.0), 1% NP40, 0.5% sodium deoxycholate, 0.1% SDS) to obtain the whole cell extract. Ten micrograms of a total protein sample was electrophoresed by a 10% SDS-PAGE gel and transferred to a PVDF membrane (Millipore, Burlington, MA, USA). The membrane was treated with blocking reagent (TBS, 0.1% Tween, 5% skim milk (Difco, Franklin Lakes, NJ, USA)), incubated with anti-VEGFR3 and subsequently with secondary antibodies conjugated with horseradish peroxidase (HRP). The signal was detected by Chemi-Lumi One L (Nacalai Tesque).

### Flow-cytometry analysis with the pERK antibody

Venus-positive and–negative cells from *Vegfr3-Gap43-Venus* BAC Tg embryos at E9.5 and were cultured in the presence of 5 μM MEK inhibitor (PD0325901; Wako, Osaka, Japan) for 30 min in a CO_2_ incubator. The cells were fixed and permeabilized with Perm Buffer III (BD Biosciences) for 30 min at 4°C and treated with the pERK antibody. FACS analysis was performed with a FACS Calibur instrument (BD Biosciences).

### Tumor implantation

Lewis lung carcinoma (LLC) cells were cultured in DMEM (BD Biosciences) containing 10% fetal bovine serum (Sigma). After the LLC cells were trypsinized, 5×10^5^ cells were suspended in D-PBS(-) (Nacalai Tesque, Kyoto, Japan) and implanted into the back of a mouse. To examine tumor angiogenesis, we implanted LLC cells in 12-week-old WT and *Vegfr3-Gap43-Venus* BAC Tg mice.

## Results

### Generating *Vegfr3-Gap43-Venus* BAC Tg mice

**Fig 1:**
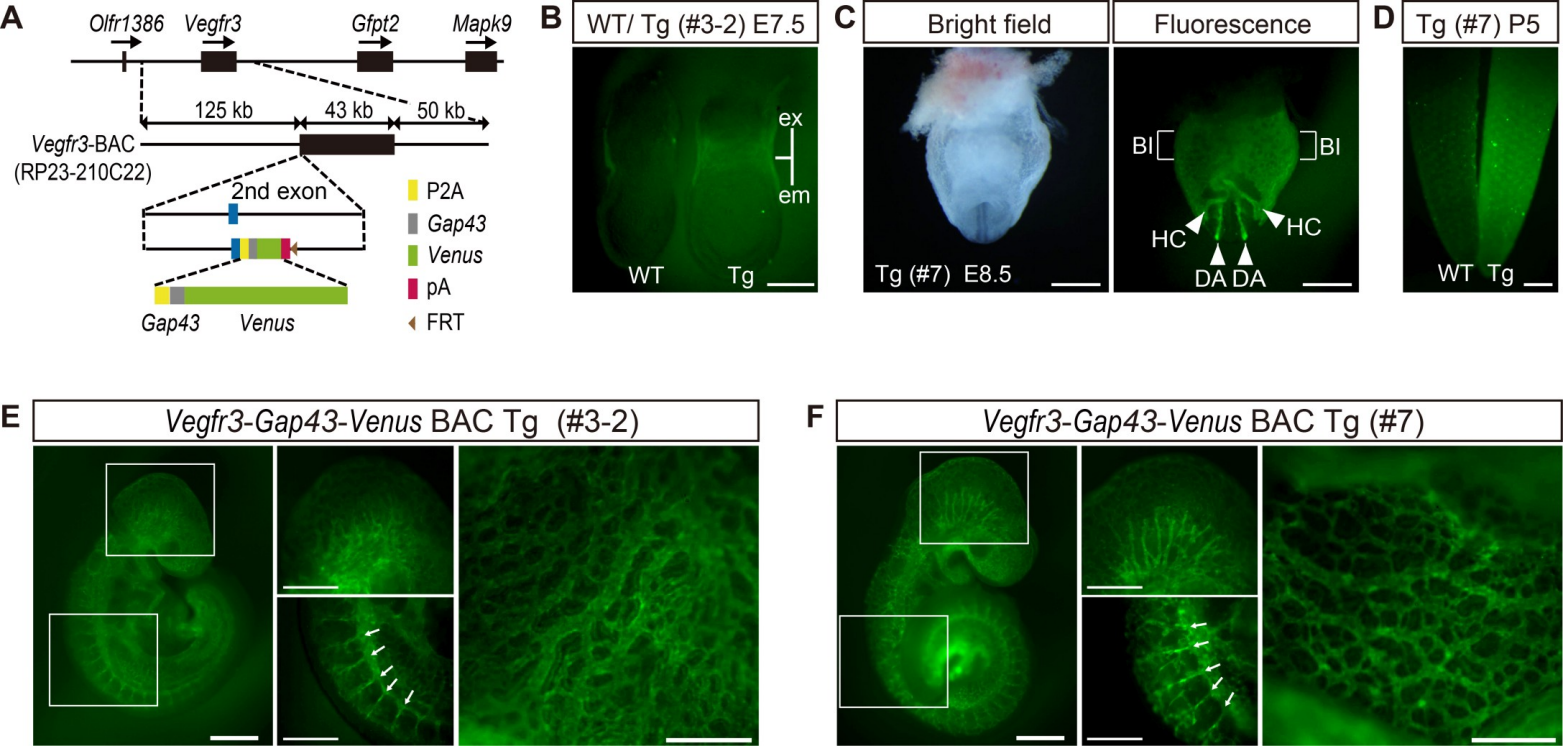
Generating *Vegfr3-Gap43-Venus* BAC Tg mice. (A) Constructing the *Vegfr3-Gap43-Venus* BAC Tg. The *Vegfr3* BAC clone (RP23-210C22) covering 125 kb upstream and 50 kb downstream of the *Vegfr3* gene was used. Note that the *PGK-gb2-neo* cassette was removed from the BAC construct prior to generating Tg mice. P2A: Pocine teschovirus self-cleaving peptide sequence, Gap43: mouse *Gap43* membrane localization sequence; pA: polyadenylation site. FRT: a recognition sequence for FLPe. (B) Venus expression in WT and Tg (line #3–2) embryos at E7.5. (C) Venus expression in Tg (line #7) embryos at E8.5. (D) Venus expression in WT and Tg (line #7) tails at P5. (E, F) Venus expression in Tg (line #3–2 and #7) embryos at E9.5. Arrows indicate intersomitic vessels. ex, extraembryonic; em, embryonic; Bl, blood island; HC, heart crescent; DA, dorsal aorta. Scale bar: 100 μm (B), 500 μm (C, D, E, F). All images were captured by a Leica FLIII microscope using GFP LP filter.

To generate transgenic (Tg) mice recapitulating endogenous membranous VEGFR3 expression, we used an enhanced yellow fluorescence protein Venus [14] with a short plasma membrane localization signal from mouse *Gap43*. We ligated the *Gap43* tagged-Venus reporter cassette to the second exon of *Vegfr3* in the BAC clone (Fig 1A), and generated four lines of *Vegfr3-Gap43-Venus* BAC Tg mice. To check Venus expression, we dissected embryos at E7.5 and E8.5 from four Tg lines and found strong Venus expression in the extraembryonic mesoderm at E7.5 and endothelial cells at E8.5 in all Tg lines (Fig 1B and 1C, data not shown), indicating that the *Vegfr3-Gap43-Venus* BAC Tg construct can efficiently direct expression in endothelial cells. Two independent *Vegfr3-Gap43-Venus* BAC Tg embryos (line# 3–2 and 7) at E9.5 showed clear Venus expression in the vascular system and these two lines were used throughout this study (Fig 1E and 1F).

### Venus expression in *Vegfr3-Gap43-Venus* BAC Tg embryos

**Fig 2:**
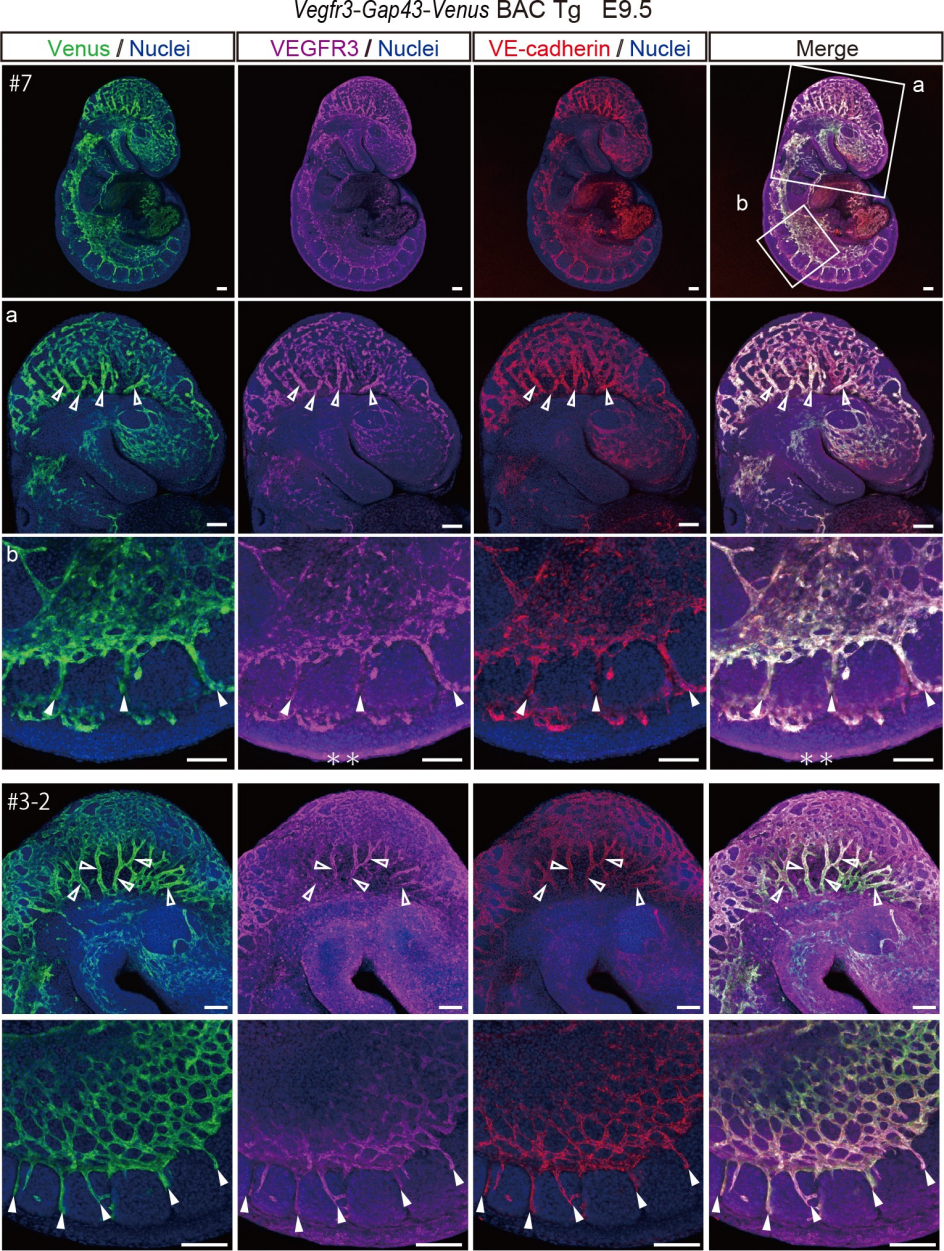
Venus expression in a *Vegfr3-Gap43-Venus* BAC Tg embryo at E9.5. Immunofluorescence analysis of a Tg embryo at E9.5 for a vascular endothelial marker, VE-cadherin (red), Venus (anti-GFP, green), VEGFR3 (magenta) and Nuclei (Hoechst33342, blue). A higher magnification of optical sections is shown in panels a and b. Note that Venus and VEGFR3 were co-expressed in the VE-cadherin–positive endothelial cells of the Tg embryo. Open and closed arrowheads indicate vascular endothelial cells in the head region and in intersomitic vessels, respectively. Scale bar: 100 μm. * indicates non-specific signal. Images were captured by a Leica TCS-SP8 confocal microscope using a 5x/0.1 dry objective lens (Upper panel), 10x/ 0.3 dry objective lens (Middle panel) and a 20x/0.7 dry objective lens (Lower panel). Immunofluorescence analysis of the Tg embryo (line# 3–2) at E9.5 for a vascular endothelial marker, VE-cadherin (red), Venus (anti-GFP, green), VEGFR3 (magenta) and Nuclei (Hoechst33342, blue). Note that Venus and VEGFR3 are co-expressed in the VE-cadherin-positive vascular endothelial cells of the Tg embryo. Open and closed arrowheads indicate endothelial cells in the head region and intersomitic vessels, respectively. Scale bar: 100 μm. Images were captured by a Leica TCS-SP8 confocal microscope using a 10x/0.15 dry objective lens (Upper panels) and 20x/0.3 dry objective lens (Lower panels).

Given the observation that endogenous VEGFR3 expression was recapitulated in the vascular system of *Vegfr3-Gap43-Venus* Tg embryos, we examined the detailed expression of Venus during embryonic development. Embryos collected at E9.5 were stained with antibodies for Venus, VEGFR3, and a vascular endothelial marker, VE-cadherin (Fig 2). Venus, VEGFR3 and VE-cadherin expressions were well-overlapped in the endothelial cells of the head region and intersomitic vessels (Fig 2).

**Fig 3:**
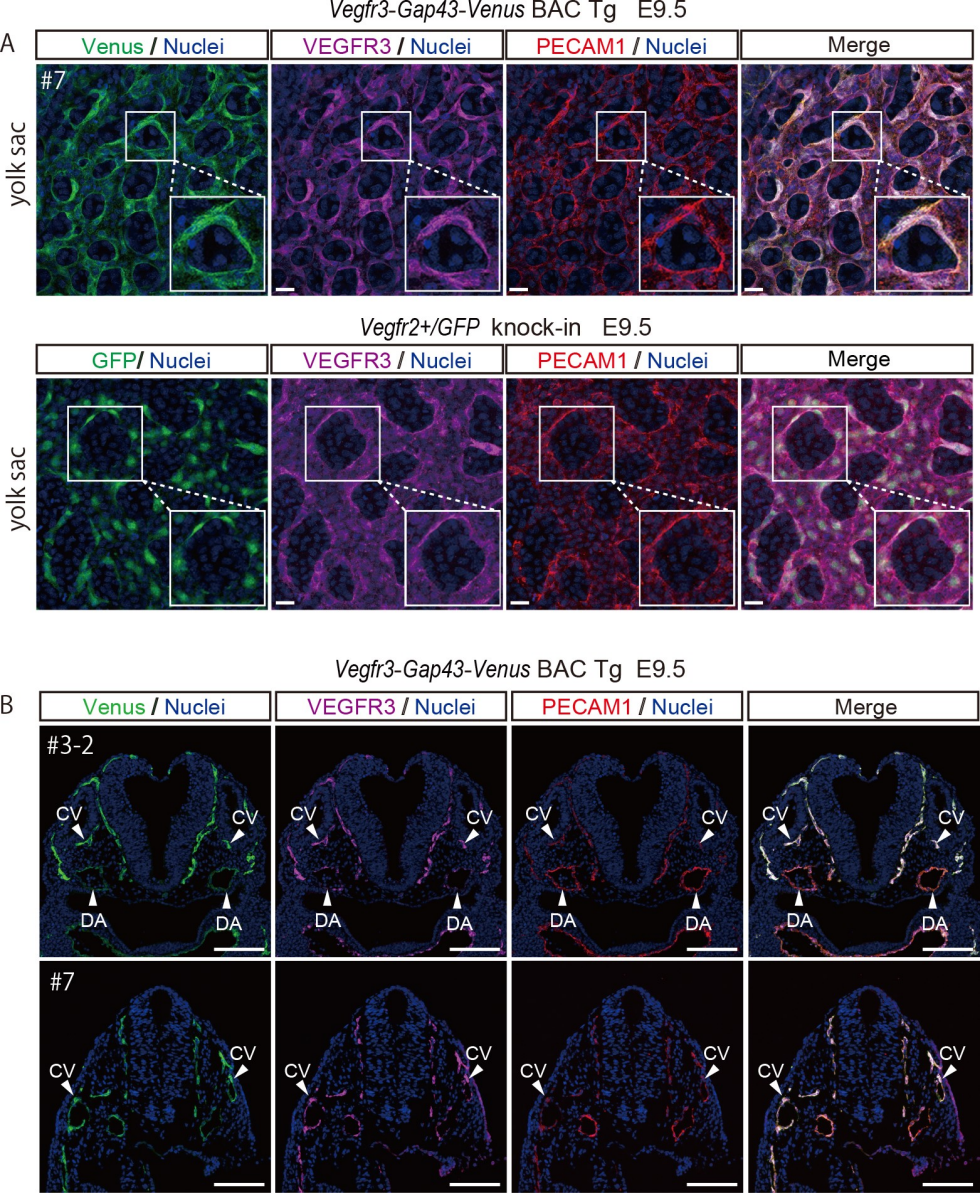
Membrane localization of Venus in a *Vegfr3-Gap43-Venus* BAC Tg yolk sac at E9.5 and Venus expression in a *Vegfr3-Gap43-Venus* BAC Tg embryo at E9.5. (A) Immunofluorescence analysis of the *Vegfr3-Gap43-Venus* BAC Tg yolk sac at E9.5 for a vascular endothelial marker, PECAM1 (red), Venus (anti-GFP, green), VEGFR3 (magenta) and Nuclei (Hoechst33342, blue). Note that the Venus of the *Vegfr3-Gap43-Venus* BAC Tg yolk sac was localized in the plasma membrane of endothelial cells, while the GFP of *Vegfr2+/GFP* knock-in was localized in the cytoplasm. Scale bar: 20 μm. All images were captured by a Leica TCS-SP8 confocal microscope using a 40x/1.25 oil objective lens. (B) Immunofluorescence images of the Tg embryo at E9.5 for PECAM1 (red), Venus (anti-GFP, green), VEGFR3 (magenta) and Nuclei (Hoechst33342, blue). Cryosections were prepared from the Tg embryos and subjected to immunohistochemistry. Note that endogenous VEGFR3 and Venus were overlapped in the vascular endothelial cells of the Tg embryo. DA: dorsal aorta; CV: cardinal vein. Scale bar: 100 μm. All images were captured by a Leica TCS-SP8 confocal microscope using a 20x/0.7 dry objective lens.

Because we used Venus with the membrane-localization signal of *Gap43*, we examined the cellular localization of Venus and found that the Gap43-Venus fluorescence pattern was very similar to that of endogenous VEGFR3 and PECAM1, which illuminate the tubular structure of blood vessels and cell shape of endothelial cells (Fig 3). In contrast, GFP without a membrane localization signal directed by *Vegfr2* was expressed in PECAM1-positive endothelial cells, but each GFP signal was intensely localized in the cytoplasm and was apparently different from those of VEGFR3 and PECAM1 (Fig 3). Thus, our *Vegfr3-Gap43-Venus* BAC Tg mouse is very useful for visualizing the cell shape and tubular structure of endothelial cells.

To confirm the overlapped pattern of Venus and VEGFR3 in more detail, cryosections were prepared and subjected to immunohistochemistry (Fig 3). Venus expression was well-overlapped with that of endogenous VEGFR3 and PECAM1 at dorsal aorta and cardinal vein. Collectively, these results demonstrated that *Vegfr3-Gap43-Venus* BAC Tg is capable of recapitulating endogenous *Vegfr3* expression in developing vascular endothelial cells.

**Fig 4:**
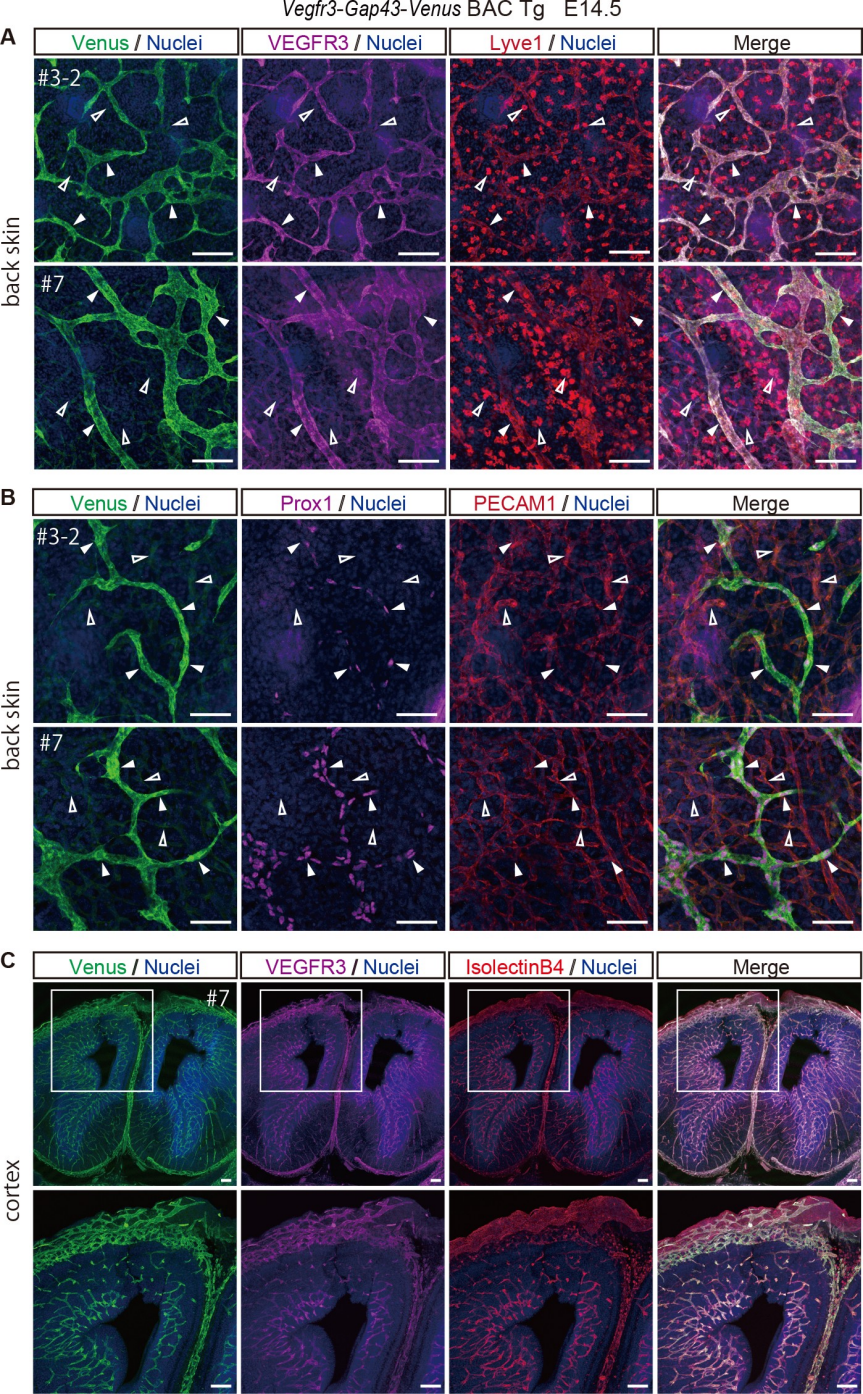
Venus expression in a *Vegfr3-Gap43-Venus* BAC Tg embryo’s skin and cortex at E14.5. (A) Immunofluorescence images of the back skin of a Tg embryo at E14.5 for Venus (anti-GFP, green), VEGFR3 (magenta), Lyve1 (red) and Nuclei (Hoechst33342, blue). Note that endogenous VEGFR3 and Venus were overlapped in the Lyve1-positive lymphatic endothelial cells of the Tg embryo. Closed arrowheads indicate Lyve1-positive lymphatic endothelial cells. Open arrowheads indicate Lyve1-positive macrophages. Scale bar: 100 μm. Images were captured by a Leica TCS-SP8 confocal microscope using a 20x/0.7 dry objective lens. (B) Immunofluorescence images of the back skin of a Tg embryo at E14.5 for Venus (anti-GFP, green), Prox1 (magenta), PECAM1 (red) and Nuclei (Hoechst33342, blue). Note that Venus was overlapped with the Prox1-positive lymphatic endothelial cells of the Tg embryo. Closed arrowheads indicate Prox1-positive lymphatic endothelial cells. Open arrowheads indicate PECAM1-positive and Prox1-negative vascular endothelial cells. Scale bar: 50 μm. All images were captured by a Leica TCS-SP8 confocal microscope using a 40x/1.25 oil objective lens. (C)Immunofluorescence images of the cortex of a Tg embryo at E14.5 for Venus (anti-GFP, green), VEGFR3 (magenta), IsolectinB4 (red) and Nuclei (Hoechst33342, blue). Note that endogenous VEGFR3 and Venus were overlapped in the IsolectinB4-stained vascular endothelial cells of the Tg embryo. Scale bar: 100 μm. Images were captured by a Leica TCS-SP8 confocal microscope using a 5x/0.15 dry objective lens (upper panels) and 10x/0.3 dry objective lens (lower panels).

Because VEGFR3 expression has been reported to be downregulated in vascular endothelial cells at the dorsal aorta, which restricts to lymphatic endothelial cells in the skin around E14.5 [5], we examined Venus expression in the back skin of E14.5 embryos and found that Venus expression was overlapped with that of endogenous VEGFR3 and Lyve1-positive lymphatic endothelial cells (Fig 4A). Prox1 -positive lymphatic endothelial cells that were integrated into tubular structures were positive for Venus (Fig 4B), indicating that Venus expression driven by *Vegfr3-Gap43-Venus* BAC recapitulates the endogenous expression of *Vegfr3* in lymphatic endothelial cells. We also examined Venus expression in the brain of *Vegfr3-Gap43-Venus* BAC Tg mice at E14.5 and E17.5 (Fig 4C, S1 Fig), and found that Venus was well-overlapped with that of endogenous VEGFR3 and was expressed in IsolectinB4-stained vascular endothelial cells (Fig 4C, S1 Fig), indicating that the timing of downregulation of VEGFR3 in vascular endothelial cells differs from organ to organ (Fig 4).

### Venus expression in *Vegfr3-Gap43-Venus* BAC Tg adult mice and in a tumor model

**Fig 5:**
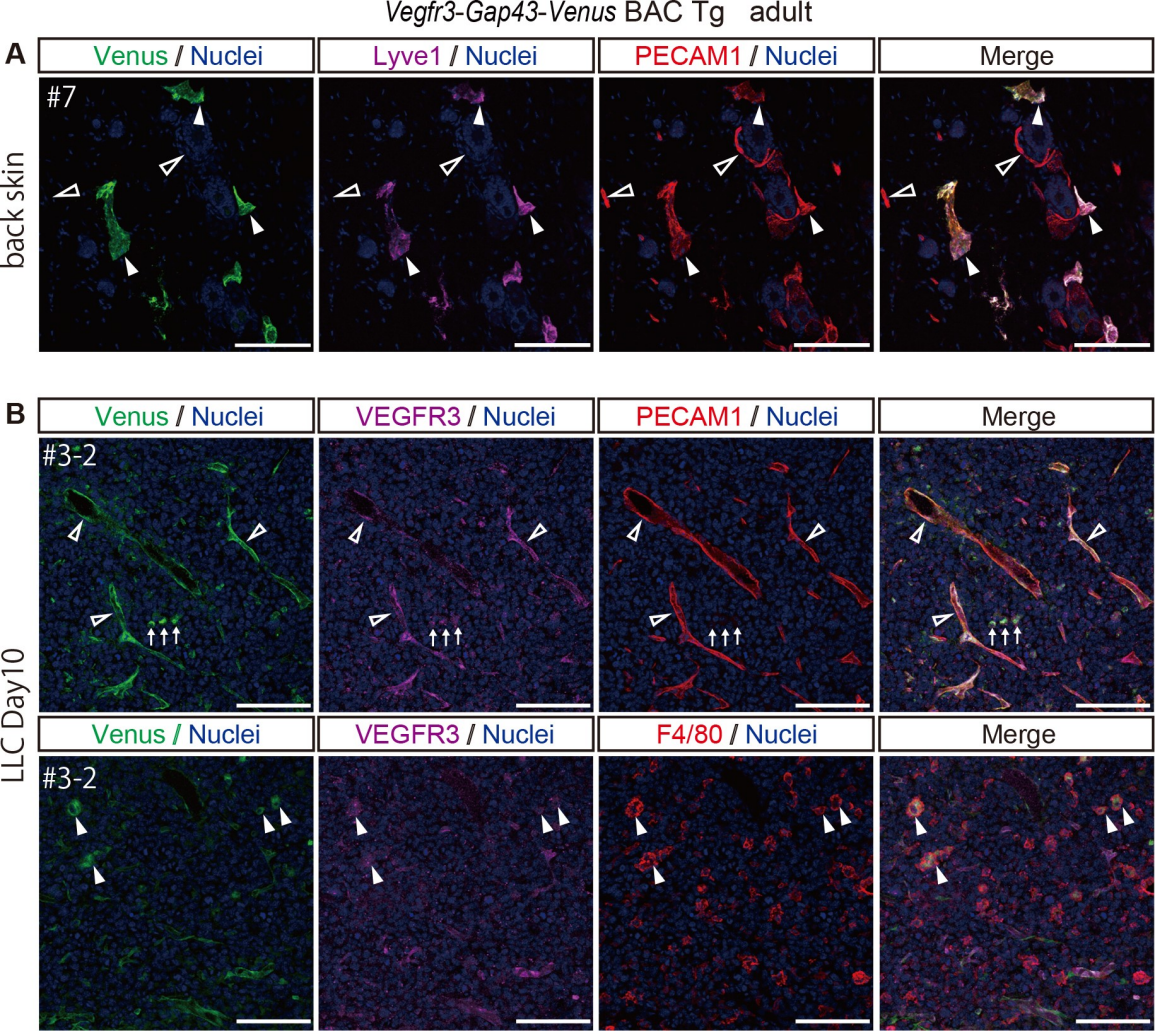
Venus expression in the normal back skin and tumor of *Vegfr3-Gap43-Venus* BAC Tg adult mice. (A) Immunofluorescence images of the back skin of an adult Tg mouse for Venus (anti-GFP, green), Lyve1 (magenta), PECAM1 (red) and Nuclei (Hoechst33342, blue). Closed and open arrowheads indicate Lyve1-positive lymphatic endothelial cells and Lyve1-negative vascular endothelial cells, respectively. (B) Immunofluorescence images of the tumor implanted in the Tg for Venus (anti-GFP, green), VEGFR3 (magenta), PECAM1 (upper panel) or F4/80 (lower panel) (red) and Nuclei (Hoechst33342, blue). Open arrowheads indicate PECAM1-positive vascular endothelial cells. Arrows indicate PECAM1-negative cells with a round morphology. Closed arrowheads indicate F4/80-positive macrophages. Scale bar: 100 μm. All images were captured by a Leica TCS-SP8 confocal microscope using a 20x/0.7 dry objective lens.

In adult mice, *Vegfr3* expression is restricted to lymphatic endothelial cells and is not detectable in most, if not all, vascular endothelial cells. However, when vascular endothelial cells invade a tumor, VEGFR3 is highly upregulated in the vascular endothelial cells and is required for tumor angiogenesis [7]. Consistent with this report, in the normal back skin of adult mice, we found that Venus was expressed predominantly in Lyve1-positive lymphatic endothelial cells, but not PECAM1-positive vascular endothelial cells (Fig 5A). However, when Lewis lung carcinoma (LLC) cells were implanted into the back skin of *Vegfr3-Gap43-Venus* BAC Tg mice, Venus was markedly upregulated in the PECAM1-positive endothelial cells in the tumor (Fig 5B). Additionally, we found Venus-positive and PECAM1-negative cells with a round morphology (Fig 5B). Previous reports showed that macrophages that infiltrate tumors express VEGFR3 and are critical for tumor progression [8,18]. Thus, we performed immunohistochemistry with a marker for macrophage, F4/80 and found that the substantial number of Venus-positive cells with a round morphology express F4/80, suggesting that they are macrophages infiltrating into the tumor (Fig 5B). Therefore, *Vegfr3-Gap43-Venus* BAC Tg is capable of recapitulating endogenous *Vegfr3* expression in adult mice and tumor model.

### Dual imaging of VEGFR3 and VEGFR1

**Fig 6:**
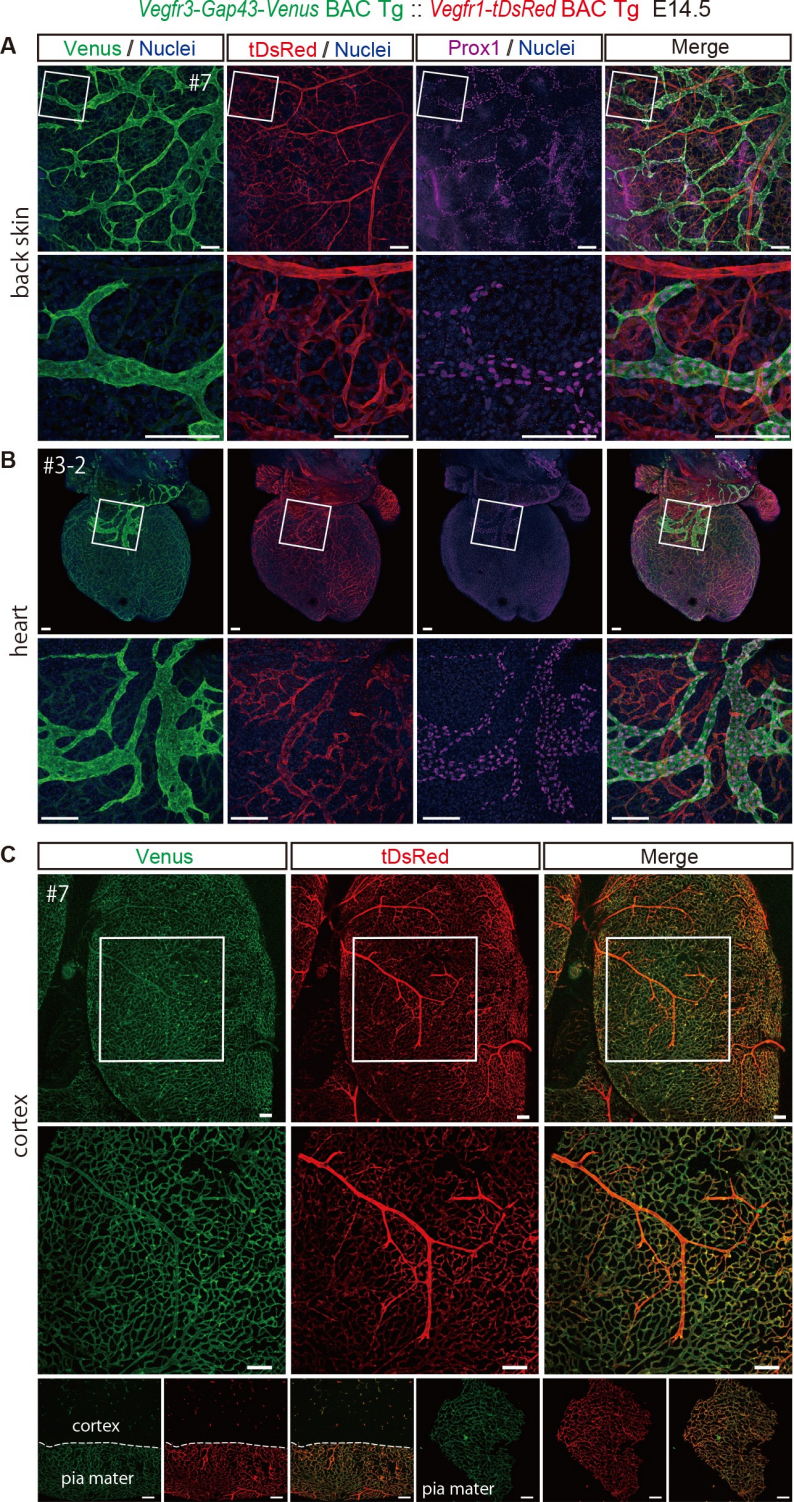
Simultaneous detection of *Vegfr3-Gap43-Venus* and *Vegfr1-tDsRed* in the skin, heart and brain cortex at E14.5. (A) Immunofluorescence images of the back skin of Tg (#7) at E14.5 embryos for Venus, tDsRed and Prox1. Note that Venus expression is overlapped with that of Prox1, but not tDsRed in vascular endothelial cells. Scale bar: 100 μm. Images were captured by a Leica TCS-SP8 confocal microscope using a 10x/0.3 dry objective lens (Upper panels) and a 40x/1.25 oil objective lens (Lower panels). (B) Frontal view of immunofluorescence images of the heart of a Tg (#3–2) mouse at E14.5 for Venus, tDsRed and Prox1. Images were captured by a Leica TCS-SP8 confocal microscope using a 5x/0.15 dry objective lens (A, B Upper panels), 10x/0.3 dry objective lens (A Lower panels), and 20x/0.7 dry objective lens (B Lower panels). (C) Top view of the brain cortex of Tg (#7) at E14.5 for Venus and tDsRed. An unstained sample treated with 2%PFA was processed for imaging. Bottom panels indicate direct fluorescence of pia mater and cortex. Scale bar: 100 μm.

Previously, we created animal models for imaging of *Vegfr1* and *Vegfr2* expression and found substantial differences between *Vegfr1* and *Vegfr2* expression patterns in embryos at 9.5 dpc; *Vegfr1* expression was more prominent in large blood vessels such as the dorsal aorta, while *Vegfr2* expression was more prominent in microcapillaries [11]. In order to investigate whether our *Vegfr3-Gap43-Venus* BAC Tg mice could be used for simultaneous imaging of Vegf Receptors, we tried to detect *Vegfr3* and *Vegfr1* expressions simultaneously. We detected clear signals of *Vegfr3-Venus* in Prox1-positive lymphatic endothelial cells and *Vegfr1-tDsRed* in vascular endothelial cells of the back skin of *Vegfr3-Gap43-Venus* BAC Tg :: *Vegfr1-tDsRed* BAC Tg embryos at E14.5 (Fig 6A, S2 Fig).

Cardiac lymphatic system arises from heterogeneous origins [19]. Thus, we investigated the expression of Venus, tDsRed and Prox1 in the heart of the Tg mouse at E14.5 (Fig 6B, S2 Fig) and found co-expression of Venus and Prox1 in the heart at E14.5. Compiled images along z-stack clearly illuminate 3D-structures of lymphatic system and coronary vasculature (S1 Movie). In the brain at E14.5, we found most, if not all, vascular endothelial cells expressed *Vegfr3*, while *Vegfr1* was expressed more strongly in larger vessels (Fig 6C, S2 Fig).

Taken together, the *Vegfr3-Gap43-Venus* BAC Tg mice established in this study is useful for studying angiogenesis and lymphangiogenesis in combination with *Vegfr1-tDsRed* BAC Tg mice.

### Quantitative colocalization analysis

**Fig 7:**
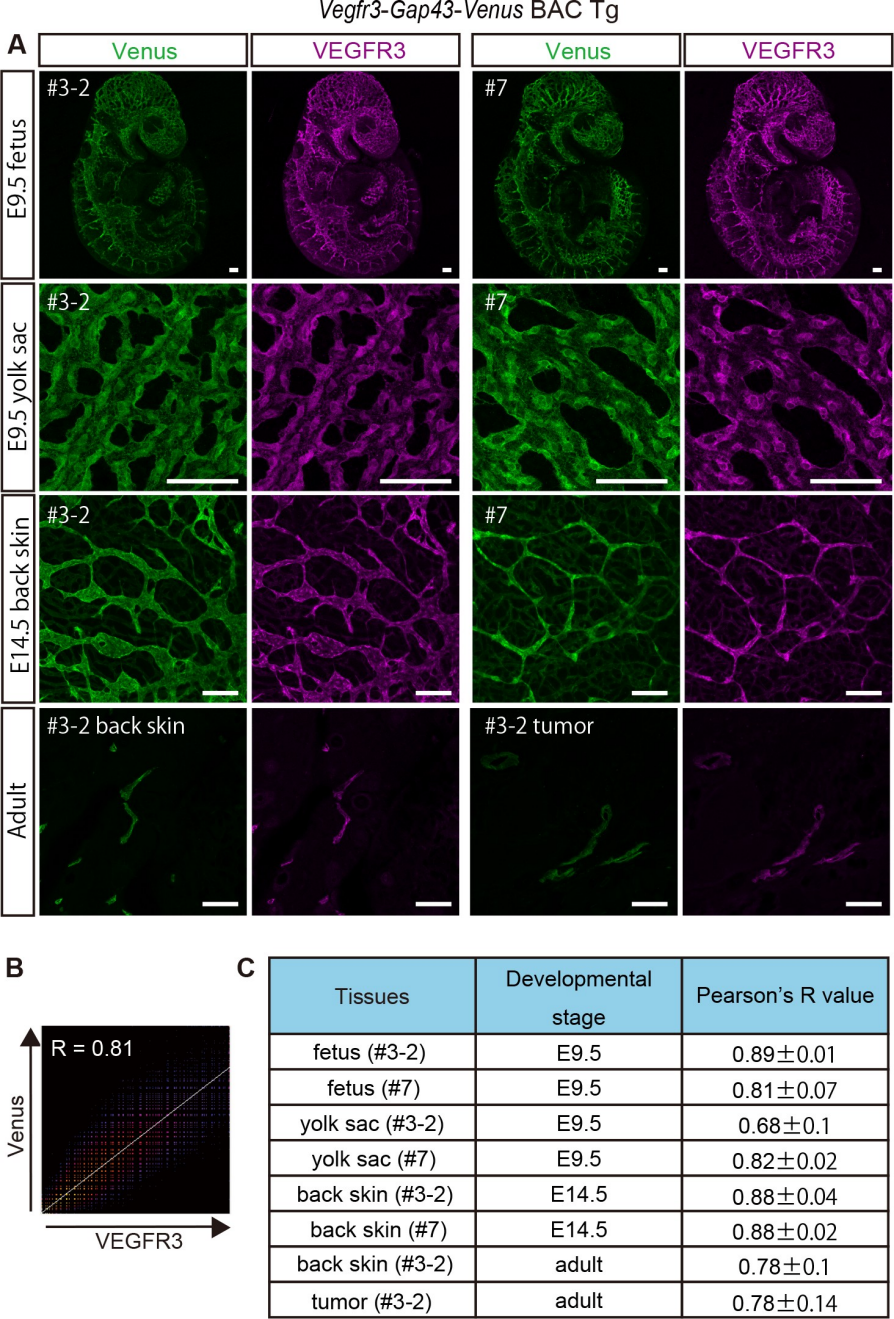
Quantitative colocalization analysis of endogenous VEGFR3 and Venus in *Vegfr3-Gap43-Venus* BAC Tg at various developmental stages. (A) Expression of endogenous VEGFR3 and Venus in Tg (#3–2, #7) at various developmental stages. (B) A 2D intensity histogram for Pearson’s *R* between VEGFR3 and Venus. (C) Summary of Pearson’s *R* between VEGFR3 and Venus at various developmental stages.

We have shown that our *Vegfr3-Gap43-Venus* BAC Tg mice recapitulate endogenous VEGFR3 expression by presenting Venus and VEGFR3 expression at various developmental stages (Figs 2–6, S1 and S2 Figs). To quantitatively examine the colocalization of Venus and VEGFR3, three independent immunostaining experiments for Venus and VEGFR3 expression were performed on E9.5, E14.5 embryos and adult skin (Fig 7), and the image data were analyzed by the ImageJ FIJI software. The analysis clearly demonstrated that the Venus reporter expression is significantly associated with the pattern of endogenous VEGFR3 (Fig 7), thus strengthening the usefulness of *Vegfr3-Gap43-Venus* BAC Tg reporter mice.

### Potential use for studying the mechanism of VEGFR3 activity

**Fig 8:**
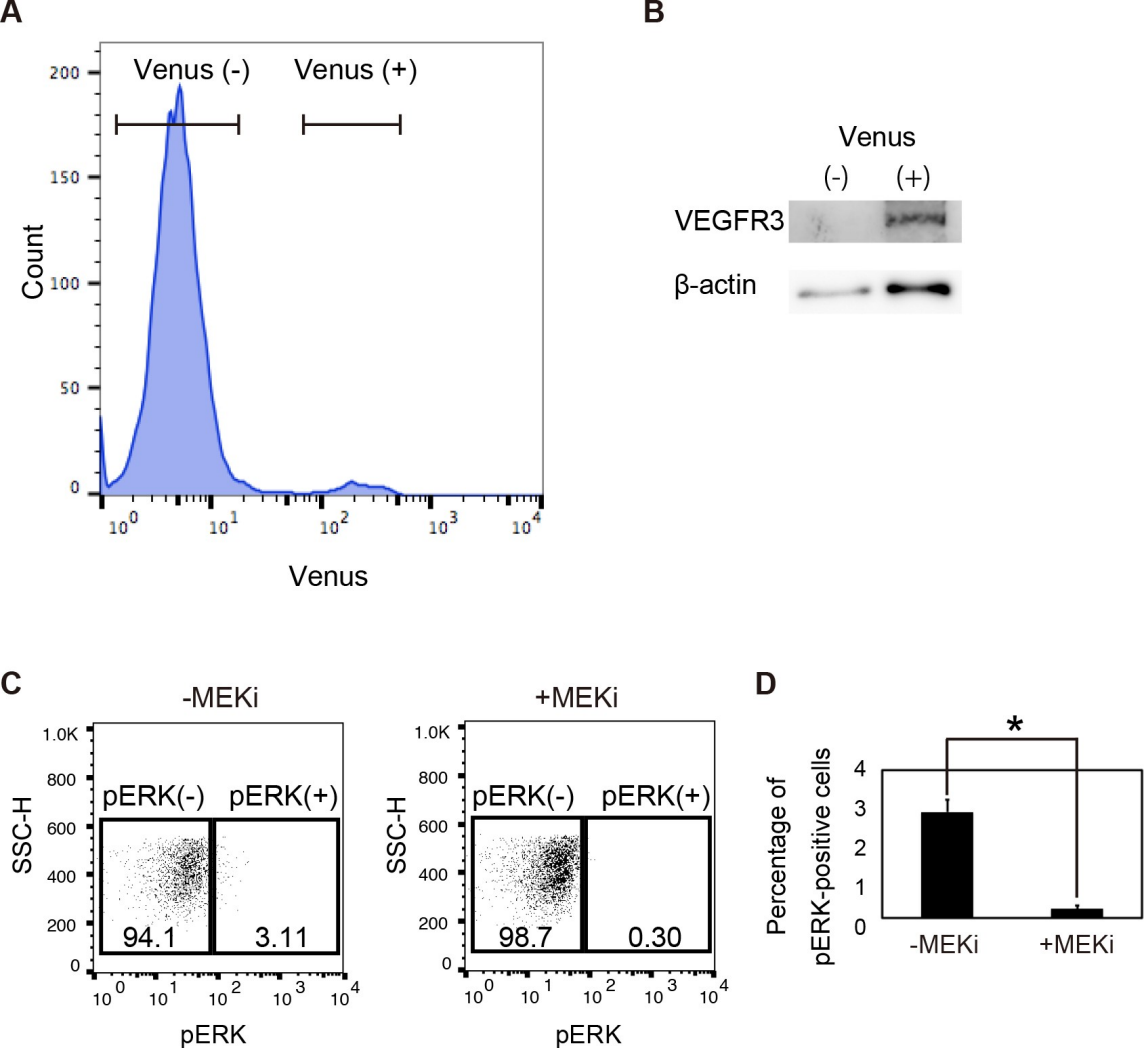
Detection of pERK signaling of vascular endothelial cells in *Vegfr3-Gap43-Venus* Tg embryos at E9.5. (A) Flow-cytometry analysis of *Vegfr3-Gap43-Venus* Tg embryos at E9.5. (B) Western blot analysis of Venus-positive and -negative cells from Tg embryos. (C) Phosphorylated ERK signaling in Venus-positive cells cultured in the presence or absence of the MEK inhibitor. (D) Percentage of pERK-positive cells in Venus-positive cells.

Although the main purpose of this study was to create a useful Vegfr3 reporter mouse to enable researchers to study angiogenesis and lymphangiogenesis, we have attempted to show that *Vegfr3-Gap43-Venus* BAC Tg mice are also valuable for studying the mechanism of VEGFR3 activity during embryonic development by molecular and biochemical analysis (Fig 8). We have sorted Venus-positive endothelial cells from E9.5 Vegfr3-Gap43-Venus BAC Tg embryos (Fig 8A) and confirmed that VEGFR3 is enriched in Venus-positive endothelial cells by western blot analysis (Fig 8B). We then showed by flow-cytometry analysis that the percentage of pERK-positive cells in Venus-positive endothelial cells is significantly reduced by MEK inhibitor treatment (Fig 8C and 8D). Thus, we have shown that our Vegfr3-Gap43-Venus BAC Tg mice can be used for studying the mechanism of VEGFR3 activity (Fig 8).

## Discussion

In this study, we demonstrated that *Vegfr3-Gap43-Venus* BAC Tg can recapitulate endogenous VEGFR3 expression in vascular endothelial cells and lymphatic endothelial cells during embryonic development and in adulthood. By using Venus with a plasma membrane localization signal, the clear tubular structure of vessels and the cell shape of endothelial cells could be visualized in *Vegfr3-Gap43-Venus* BAC Tg mice. Overall, Venus expression was much stronger and clearer with less background signal than that of antibody staining against VEGFR3. In tumor tissue, it is generally difficult to detect endogenous gene expression, including VEGFR3, by immunohistochemistry. In contrast, *Vegfr3-*Venus is bright and detectable easily as described in our study, and will thus be very useful for studying angiogenesis during tumorigenesis.

Simultaneous imaging of vascular endothelial cells and lymphatic endothelial cells is useful for studying angiogenesis and lymphangiogenesis. Azar and coworker established *Prox1-GFP* :: *Flt1-tDsRed* Tg mice for simultaneous imaging of angiogenesis and lymphangiogenesis [20]. We demonstrated that the *Vegfr3-Gap43-Venus* :: *Vegfr1-tDsRed* BAC Tg mice established in this study can be used to visualize developing vascular endothelial cells in the brain, lymphatic endothelial cells in the heart, and in other areas. Furthermore, in contrast with *Prox1-GFP*, our Tg utilizes a Venus with Gap43 membrane localization signal and has an advantage to illuminate cell shape of endothelial cells such as tip cells.

VEGF-C binds to VEGFR2/VEGFR3 heterodimer and activates Akt signaling, whereas VEGFR3/VEGFR3 homodimer activates ERK signaling [3]. It is important to distinguish cells expressing VEGFR2 alone, VEGFR3 alone or VEGFR2/VEGFR3. For that purpose, we established *Vegfr3-Gap43-Venus* :: *Vegfr2+/GFP* knock-in mice and tried to distinguish Venus and GFP fluorescence. However, close excitation and emission wavelengths between Venus and GFP, and the strong fluorescent intensity of *Vegfr2*-GFP over *Vegfr3*-Venus hampered dual detection of the *Vegfr2*-GFP and *Vegfr3*-Venus signal (C. W., M. E. unpublished observation). In the future, red fluorescent protein in *Vegfr3* BAC Tg or cyan fluorescent protein in *Vegfr2* BAC Tg mice will be needed for dual imaging of VEGFR2 and VEGFR3.

Previous studies have shown that *Vegfr3* is expressed in vascular endothelial and lymphatic endothelial cells [5], neural stem cells [21], macrophages and osteoblasts [8,9], and it is important for vascular development [21], lymphangiogenesis [6] and neural stem cells [9]. Therefore, it is very important to understand exactly where and at what level it is expressed. Taken together, the *Vegfr3-Gap43-Venus* BAC Tg mice established in our study may be used to investigate novel functions of *Vegfr3* as well as to study tumor angiogenesis.

## Supporting information

S1 Table List for first and second antibodies used in this study. Antibodies used in this study are presented.
(PDF)

S1 Fig Venus expression in the brain of a *Vegfr3-Gap43-Venus* BAC Tg embryo. Immunofluorescence images of the brain cortex of a Tg (#3–2) embryo at E17.5 for Venus (anti-GFP, green), VEGFR3 (red), IsolectinB4 (magenta) and Nuclei (Hoechst33342, blue). Note that endogenous VEGFR3 and Venus were overlapped in the IsolectinB4-stained vascular endothelial cells of the Tg embryo. Scale bar: 100 μm. All images were captured by a Leica TCS-SP8 confocal microscope using a 20x/0.7 dry objective lens.
(TIF)

S2 Fig Simultaneous detection of *Vegfr3-Gap43-Venus* and *Vegfr1-tDsRed* in the skin, heart and brain cortex at E14.5. (A) Immunofluorescence images of the back skin of the Tg (#3–2) at E14.5 for Venus (anti-GFP, green), tDsRed (red) and Prox1 (magenta). Note that Venus is overlapped with Prox1, but not tDsRed in vascular endothelial cells. Scale bar: 100 μm. Images were captured by a Leica TCS-SP8 confocal microscope using a 20x/0.7 dry objective lens (Upper panels) and a 40x/1.25 oil objective lens (Lower panels). (B) Frontal view of immunofluorescence images of the heart of a Tg (#7) mouse at E14.5 for Venus (anti-GFP, green), tDsRed (red) and Prox1 (magenta). Images were captured by a Leica TCS-SP8 confocal microscope using a 10x/0.3 dry objective lens (A), 5 x 0.15 dry objective lens (B Upper panels) and 20x/0.7 dry objective lens (B Lower panels). (C) Top view of the brain cortex of Tg (#3–2) at E14.5 for Venus and tDsRed. An unstained sample was processed for imaging. Scale bar: 100 μm.
(TIF)

S1 Movie 3D image of frontal view of immunofluorescence images of the heart of a Tg (#3–2) mouse at E14.5 for Venus (anti-GFP, green) and tDsRed(red). Images were captured by a Leica TCS-SP8 confocal microscope using a 20x/0.7 dry objective lens.
(MOV)

## References

[1] Shibuya M, Claesson-Welsh L. Signal transduction by VEGF receptors in regulation of angiogenesis and lymphangiogenesis. Exp Cell Res. 2006;312: 549–60. 10.1016/j.yexcr.2005.11.012 16336962

[2] Matsumoto K, Ema M. Roles of VEGF-A signalling in development, regeneration, and tumours. J Biochem. 2014;156: 1–10. 10.1093/jb/mvu031 24839295

[3] Deng Y, Zhang X, Simons M. Molecular controls of lymphatic VEGFR3 signaling. Arterioscler Thromb Vasc Biol. 2015;35: 421–9. 10.1161/ATVBAHA.114.304881 25524775 PMC4304921

[4] Ferrara N, Gerber H-P, LeCouter J. The biology of VEGF and its receptors. Nat Med. 2003;9: 669–76. 10.1038/nm0603-669 12778165

[5] Kaipainen A, Korhonen J, Mustonen T, van Hinsbergh VW, Fang GH, Dumont D, et al Expression of the fms-like tyrosine kinase 4 gene becomes restricted to lymphatic endothelium during development. Proc Natl Acad Sci U S A. 1995;92: 3566–70. Available: http://www.ncbi.nlm.nih.gov/pubmed/7724599 7724599 10.1073/pnas.92.8.3566 PMC42208

[6] Alitalo K. The lymphatic vasculature in disease. Nat Med. 2011;17: 1371–80. 10.1038/nm.2545 22064427

[7] Tammela T, Zarkada G, Wallgard E, Murtomäki A, Suchting S, Wirzenius M, et al Blocking VEGFR-3 suppresses angiogenic sprouting and vascular network formation. Nature. 2008;454: 656–60. 10.1038/nature07083 18594512

[8] Koch S, Tugues S, Li X, Gualandi L, Claesson-Welsh L. Signal transduction by vascular endothelial growth factor receptors. Biochem J. 2011;437: 169–83. 10.1042/BJ20110301 21711246

[9] Calvo C-F, Fontaine RH, Soueid J, Tammela T, Makinen T, Alfaro-Cervello C, et al Vascular endothelial growth factor receptor 3 directly regulates murine neurogenesis. Genes Dev. 2011;25: 831–44. 10.1101/gad.615311 21498572 PMC3078708

[10] Ishitobi H, Matsumoto K, Azami T, Itoh F, Itoh S, Takahashi S, et al Flk1-GFP BAC Tg mice: an animal model for the study of blood vessel development. Exp Anim. 2010;59: 615–22. Available: http://www.ncbi.nlm.nih.gov/pubmed/21030789 21030789 10.1538/expanim.59.615

[11] Matsumoto K, Azami T, Otsu A, Takase H, Ishitobi H, Tanaka J, et al Study of normal and pathological blood vessel morphogenesis in Flt1-tdsRed BAC Tg mice. Genesis. 2012;50: 561–71. 10.1002/dvg.22031 22489010

[12] Ishitobi H, Wakamatsu A, Liu F, Azami T, Hamada M, Matsumoto K, et al Molecular basis for Flk1 expression in hemato-cardiovascular progenitors in the mouse. Development. 2011;138: 5357–68. 10.1242/dev.065565 22071109 PMC4074304

[13] Okabe K, Kobayashi S, Yamada T, Kurihara T, Tai-Nagara I, Miyamoto T, et al Neurons limit angiogenesis by titrating VEGF in retina. Cell. 2014;159: 584–96. 10.1016/j.cell.2014.09.025 25417109

[14] Nagai T, Ibata K, Park ES, Kubota M, Mikoshiba K, Miyawaki A. A variant of yellow fluorescent protein with fast and efficient maturation for cell-biological applications. Nat Biotechnol. 2002;20: 87–90. 10.1038/nbt0102-87 11753368

[15] Ema M, Takahashi S, Rossant J. Deletion of the selection cassette, but not cis-acting elements, in targeted Flk1-lacZ allele reveals Flk1 expression in multipotent mesodermal progenitors. Blood. 2006;107: 111–7. 10.1182/blood-2005-05-1970 16166582

[16] Ema M, Yokomizo T, Wakamatsu A, Terunuma T, Yamamoto M, Takahashi S. Primitive erythropoiesis from mesodermal precursors expressing VE-cadherin, PECAM-1, Tie2, endoglin, and CD34 in the mouse embryo. Blood. 2006;108: 4018–24. 10.1182/blood-2006-03-012872 16926294

[17] Schindelin J, Arganda-Carreras I, Frise E, Kaynig V, Longair M, Pietzsch T, et al Fiji: An open-source platform for biological-image analysis. Nat Methods. 2012;9: 676–682. 10.1038/nmeth.2019 22743772 PMC3855844

[18] Alishekevitz D, Gingis-Velitski S, Kaidar-Person O, Gutter-Kapon L, Scherer SD, Raviv Z, et al Macrophage-Induced Lymphangiogenesis and Metastasis following Paclitaxel Chemotherapy Is Regulated by VEGFR3. Cell Rep. 2016;17: 1344–1356. 10.1016/j.celrep.2016.09.083 27783948 PMC5098117

[19] Klotz L, Norman S, Vieira JM, Masters M, Rohling M, Dubé KN, et al Cardiac lymphatics are heterogeneous in origin and respond to injury. Nature. 2015;522: 62–7. 10.1038/nature14483 25992544 PMC4458138

[20] Zhong W, Gao X, Wang S, Han K, Ema M, Adams S, et al Prox1-GFP/Flt1-DsRed transgenic mice: an animal model for simultaneous live imaging of angiogenesis and lymphangiogenesis. Angiogenesis. 2017;20: 581–598. 10.1007/s10456-017-9572-7 28795242 PMC5782821

[21] Dumont DJ, Jussila L, Taipale J, Lymboussaki A, Mustonen T, Pajusola K, et al Cardiovascular failure in mouse embryos deficient in VEGF receptor-3. Science. 1998;282: 946–9. Available: http://www.ncbi.nlm.nih.gov/pubmed/9794766 9794766 10.1126/science.282.5390.946

