# Supplementary material for: Generating Vegfr3 reporter transgenic mouse expressing membrane-tagged Venus for visualization of VEGFR3 expression in vascular and lymphatic endothelial cells
Source: PLoS One. 2019 Jan 2;14(1):e0210060. doi: 10.1371/journal.pone.0210060 (PMC6314617; doi:10.1371/journal.pone.0210060)
Supplement: S1 Table — Antibodies used in this study are presented. (PDF) [file pone.0210060.s001.pdf]

List of antibodies

Primary Antibody	Supplier	Catalogue Number	Dilution
Alexa Fluor 488 rabbit anti-GFP	Thermo Fisher Scientific	A-21311	1:200
Rat anti-CD31	BD Biosciences	553370	1:100
Rat anti-VE-cadherin	BioLegend	138101	1:100
Rabbit anti-LYVE1	abcam	ab14917	1:400
Goat anti-Prox1	R&D Systems	AF2727	1:50
Goat anti-VEGFR3	R&D Systems	AF743	1:200
Rabbit anti-RFP	MBL	PM005	1:200
PE Mouse Anti-ERK1/2 (pT202/pY204)	BD Biosciences	612566	1:6
Anti-β-Actin pAb-HRP-DirecT	MBL	PM053-7	1:2000
HRP-conjugated donkey anti-Goat	Jackson ImmunoResearch	705-035-003	1:5000

Secondary Antibody	Supplier	Catalogue Number	Dilution
Cy3-conjugated donkey anti-rat IgG	Jackson ImmunoResearch	712-165-150	1:400
Cy3-conjugated donkey anti-rabbit IgG	Jackson ImmunoResearch	711-165-152	1:400
Cy5-conjugated donkey anti-rat IgG	Jackson ImmunoResearch	712-175-153	1:400
Alexa633-conjugated donkey anti-goat IgG	Thermo Fisher Scientific	A21082	1:400
Alexa647-conjugated donkey anti-goat IgG	Thermo Fisher Scientific	A21447	1:400

Isolectin GS-IB4 Alexa Fluor 647 Conjugate	Thermo Fisher Scientific	I32450	1:100
Hoechst 33342 (10mg/ml)	Thermo Fisher Scientific	H3570	1:1000

S1 Table
