# Supplementary figures and images for: Generating Vegfr3 reporter transgenic mouse expressing membrane-tagged Venus for visualization of VEGFR3 expression in vascular and lymphatic endothelial cells
Source: PLoS One. 2019 Jan 2;14(1):e0210060. doi: 10.1371/journal.pone.0210060 (PMC6314617; doi:10.1371/journal.pone.0210060)

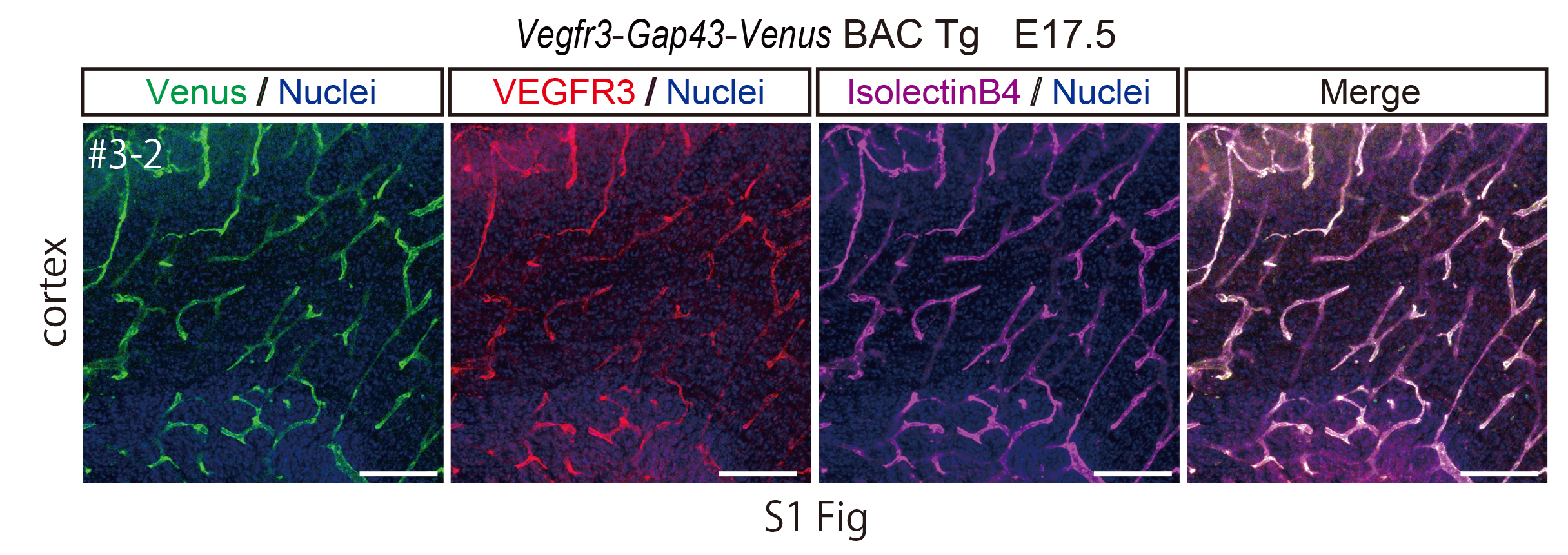

Supplement: S1 Fig — Immunofluorescence images of the brain cortex of a Tg (#3–2) embryo at E17.5 for Venus (anti-GFP, green), VEGFR3 (red), IsolectinB4 (magenta) and Nuclei (Hoechst33342, blue). Note that endogenous VEGFR3 and Venus were overlapped in the IsolectinB4-stained vascular endothelial cells of the Tg embryo. Scale bar: 100 μm. All images were captured by a Leica TCS-SP8 confocal microscope using a 20x/0.7 dry objective lens. (TIF) [file pone.0210060.s002.tif]

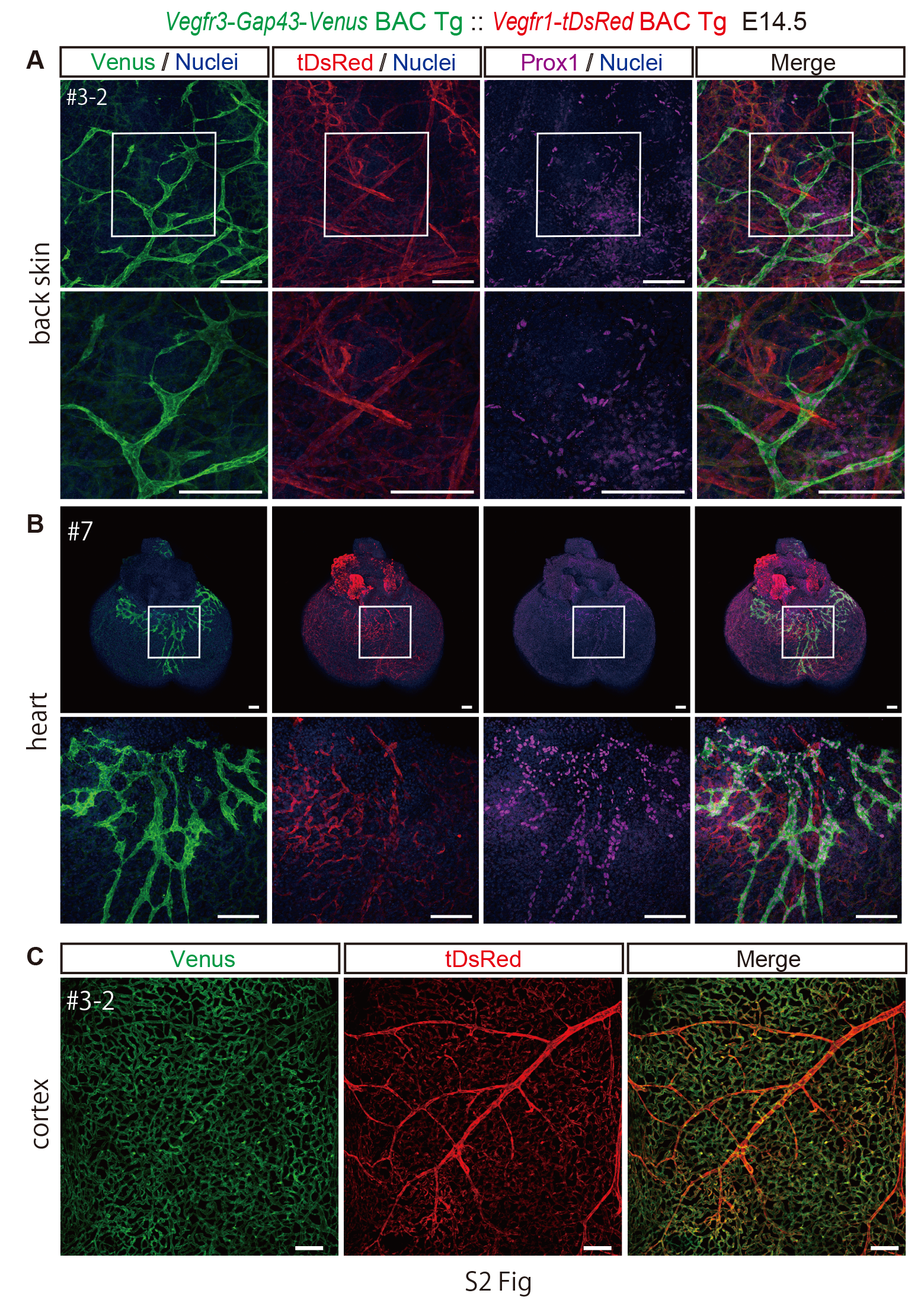

Supplement: S2 Fig — (A) Immunofluorescence images of the back skin of the Tg (#3–2) at E14.5 for Venus (anti-GFP, green), tDsRed (red) and Prox1 (magenta). Note that Venus is overlapped with Prox1, but not tDsRed in vascular endothelial cells. Scale bar: 100 μm. Images were captured by a Leica TCS-SP8 confocal microscope using a 20x/0.7 dry objective lens (Upper panels) and a 40x/1.25 oil objective lens (Lower panels). (B) Frontal view of immunofluorescence images of the heart of a Tg (#7) mouse at E14.5 for Venus (anti-GFP, green), tDsRed (red) and Prox1 (magenta). Images were captured by a Leica TCS-SP8 confocal microscope using a 10x/0.3 dry objective lens (A), 5 x 0.15 dry objective lens (B Upper panels) and 20x/0.7 dry objective lens (B Lower panels). (C) Top view of the brain cortex of Tg (#3–2) at E14.5 for Venus and tDsRed. An unstained sample was processed for imaging. Scale bar: 100 μm. (TIF) [file pone.0210060.s003.tif]
